# The North American opioid epidemic: current challenges and a call for treatment as prevention

**DOI:** 10.1186/s12954-017-0135-4

**Published:** 2017-05-12

**Authors:** Devesh Vashishtha, Maria Luisa Mittal, Daniel Werb

**Affiliations:** 1 Division of Global Public Health, Department of Medicine, University of California, San Diego, 9500 Gilman Drive, La Jolla, CA 92093-0507 USA; 2 Facultad de Medicina, Universidad Xochicalco Campus Tijuana, RampaYumalinda 4850, Colonia Chapultepec Alamar C.P.22540, Tijuana, Baja California México

**Keywords:** Opioid epidemic, North America, Treatment as Prevention, Injection initiation, People who inject drugs, Medication-assisted treatment

## Abstract

There is a need for creative, public health-oriented solutions to the increasingly intractable problems associated with the North American opioid epidemic. This epidemic is a fundamentally continental problem, as routes of migration, drug demand, and drug exchange link the USA with Mexico and Canada. The challenges faced throughout North America include entrenched prescribing practices of opioid medications, high costs and low availability of medication-assisted treatment (MAT), and policy approaches that present substantial barriers to care.

We advocate for the scale up of a low-threshold treatment model for MAT that incorporates the best practices in addiction treatment. Such a model would remove barriers to care through widespread treatment availability and affordability and also a policy of decriminalization. Given that MAT reduces the frequency of drug injecting among opioid injectors, this treatment model should also be guided by an understanding of the socially communicable nature of injection drug use, such that increasing MAT availability may also prevent the spread of injecting practices to individuals at risk of transitions from non-injection to injection drug use. To that end, the “Treatment as Prevention” model employed to respond to the individual- and population-level risks for HIV/AIDS prevention could be adapted to efforts to halt the North American opioid epidemic.

## Background

North America is in the midst of a massive opioid misuse epidemic. In the USA, over 2.4 million people meet the criteria for severe opioid use disorder (OUD) involving dependence on opioid analgesic medications, heroin, or both [1]. Since 2013, deaths from drug overdose have surpassed deaths from motor vehicle accidents, making overdose the leading cause of preventable death in the USA [2]. In Canada, as of 2012, there are an estimated 75,000 to 125,000 people who inject drugs (PWID) and an estimated 200,000 individuals dependent on prescription opioids [3]. In Mexico, there are over 100,000 persons who use opioids, and from 2002 to 2008 there was an increasing number of heroin users [4]. While the opioid epidemic has its roots in prescribing patterns in the USA and Canada [5], more recently, it has become a continental issue involving migration, drug trafficking networks, and patterns of drug use that occur across borders. As such, the dynamics of the epidemic in the USA are inextricably linked to events and patterns of trafficking and use in Canada and Mexico.

Efforts to halt the opioid epidemic have varied across the countries in North America. In Mexico, the government placed access to medication-assisted treatment (MAT) at the center of their policy response, while in Canada there have been multiple successful efforts to expand access to treatment for marginalized populations [4, 6]. In November 2016, the U.S. Congress voted to fund former President Obama’s 1-billion-dollar Comprehensive Addiction and Recovery Act (CARA) [7]. This proposal to address the opioid crisis focuses on increased access to MAT in the form of methadone, buprenorphine, and injectable naltrexone, and will target funding to states that are most affected by the opioid epidemic and have innovative plans to address treatment disparities [8].

As a clinical research team, we maintain that the evolving response to the crisis of OUD must prioritize the provision of MAT. In this commentary, we review current challenges in responding to opioid misuse, describe barriers to the treatment of OUD through MAT, and explore public health-oriented policy and interventional options to effectively respond to OUD in North America.

## Current challenges in opioid misuse prevention and treatment

### Prescribing practices

In the USA, pharmaceutical advertising and prescription practices have undoubtedly contributed to the current opioid epidemic [9]. With Surgeon General Vivek Murthy’s recent letter to America’s doctors, the “Turn the Tide” initiative, and the first ever Surgeon General’s Report on Alcohol, Drugs, and Health [10], opioid prescribing practices have become a high-profile issue in the USA [11]. Prescribing guidelines from the CDC [12] emphasize that opioid prescriptions are to generally be avoided for chronic non-malignant (i.e., non-cancer) pain, and that if an opioid is deemed necessary, providers should “start low and go slow” [11]. While this is an excellent starting point, it is also likely that providers will take many years to fully adapt to the new guidelines and that substantial training in pain management and addiction medicine is required.

### MAT infrastructure and cost

For persons suffering from OUD, MAT remains the clinical gold standard for treatment [10]. In the USA, however, there is inadequate infrastructure for MAT delivery [1] and a treatment gap exists, with more than 1 million eligible opioid-dependent individuals not receiving care [1]. Despite the well-established cost-effectiveness of MAT, many methadone maintenance programs in the USA have also been closed because of lack of funding, and clinicians have been discouraged from establishing MAT because compliance with federal methadone regulations is too time-consuming in a private practice model [13].

Marginalized populations across North America experience a range of barriers to being prescribed MAT. In Mexico, an intake diagnostic package fee must be made before patients can be enrolled in long-term methadone therapy [14]. In the USA, new MAT treatments including buprenorphine are often not covered by insurance [1, 15]. Part of the treatment gap in the USA is likely attributable to the lack of a national healthcare system, which has allowed for the scale up of buprenorphine prescribing in France and Canada [6, 16]. Further, in general, the USA is lacking in low-threshold programs, which increase treatment accessibility for the greatest number of individuals in need [6]. Low-threshold models might involve free or low-cost therapy, shortened waiting lines, and integrated care centers that provide mental health services. Such models are becoming the standard of care in countries such as Canada and elsewhere [6]. In Vancouver, Canada, for example, methadone is dispensed at pharmacies and integrated mental health treatment, and social support services for pregnant opioid users have been implemented [6]. In the USA, there have been successful examples of low-threshold programs such as the San Francisco Department of Public Health’s office-based Buprenorphine Pilot Program, aimed at integrating buprenorphine treatment into the outpatient setting [17], as well as the office-based buprenorphine model piloted through community health centers in Massachusetts [18, 19]. However, these programs are rare and often remain within the pilot phase in the USA [6]. There are also significant disparities in access to MAT by race, as white patients tend to receive buprenorphine while black and Latino patients are more likely to receive methadone [20].

### Provider-level factors

Building upon the U.S. Health and Human Services Opioid Initiative, the Substance Abuse and Mental Health Services Administration (SAMHSA) will expand MAT availability by allowing previously trained nurse practitioners and physician assistants to prescribe MAT in the form of buprenorphine in early 2017 [21]. However, this new low-threshold model might face challenges in implementation, as is the case with physicians, of whom only 2.2% are waivered to provide buprenorphine [15]. Physicians have also been characterized as having “low confidence in addressing addiction, limited access to addiction experts, lack of institutional or office support, lack of behavioral health services, and reimbursement concerns” [1]. This is related to the fact that physicians receive little addiction training and have ongoing stigma against treating PWID [22–24]. In fact, the lack of experience with addiction treatment in the USA is thought to be a primary barrier to buprenorphine prescribing in the USA [25].

Mexico faces an even more serious challenge, as primary care physicians are unable to directly prescribe MAT to patients. This is because methadone treatment is the only MAT option available in Mexico, and it is only dispensed in a few private clinics, while only three government-sponsored clinics are in operation across the entire country [4]. Given the high prevalence of opioid use in Mexico’s northern border region, leading health authorities in the country have therefore called for a national scale up of methadone treatment [4].

### Policy factors

In the USA, despite the fact that President Obama’s 1-billion-dollar bill to address the opioid addiction crisis was fully funded with bipartisan support in November of 2016 [7], there remains ongoing stigma in the USA against opioid treatment programs, which has resulted in numerous state and local-level policy barriers to care [1]. In Mexico, despite the 2009 drug policy reform aimed to increase the engagement of OUD individuals in MAT by simultaneously decriminalizing drug possession and having the criminal justice system divert individuals into addiction treatment, rates of MAT enrollment remain low due to high cost, inadequate insurance coverage, a lack of knowledge of the law, and low MAT scale up [26].

### Law enforcement-related factors

Street-level drug market policing remains a key barrier to addiction treatment access in North America among marginalized drug-using populations [27]. In Mexico, certain policing practices such as active surveillance, police sweeps, and extortion hinder MAT retention and reduce the financial capacity of PWID to cover MAT visit payments [27]. Policing practices in Mexico are therefore the subject of an ongoing police education program, which focuses on HIV prevention through various means including MAT provision [28]. The USA has punitive illegal drug policies that are likely contributing to high levels of illegal drug use [27], and it has been suggested that a focus on supply-side drug market interventions in the USA has not meaningfully impacted the availability of illegal drugs; indeed, over the past decades, there has been an increase in the purity and a decrease in the price of drugs including cannabis, cocaine, and heroin [29, 30]. This is particularly concerning given President-Elect Donald Trump’s recent comment that his solution to the heroin epidemic would be to “cut off the source, build a wall” [31]. While similar outcomes have been demonstrated in Canada, emerging ad hoc public health and policing partnerships, including police diversion of PWID to medically supervised injection facilities, suggest a potential role for police in improving the engagement of individuals with OUD in appropriate care [27].

## Future directions

While many challenges exist in developing an effective and comprehensive treatment system for persons with OUD in North America, concrete steps, led by clinicians [32], should be taken to bridge the treatment gap and control the opioid epidemic.

### A public health-oriented treatment system

First, federal and state-level funding for MAT treatment centers must be increased to address the 92% of opioid-dependent individuals eligible for MAT treatment [33]. In the USA, this will require building on momentum from the funding of former President Obama’s 1-billion-dollar proposal [8].

Second, barriers that hamper the capacity of clinicians to prescribe MAT must be removed. In countries such as Slovenia, Croatia, and Switzerland, the widespread availability of methadone and buprenorphine has contributed to very low HIV prevalence among injecting drug users [6]. In France, all physicians can prescribe buprenorphine without any waiver limits [6]. If MAT can be provided in physicians’ offices, patient visits could then also provide an opportunity to address comorbidities associated with opioid use including HIV and Hepatitis C. Further, in Canada, MAT dispensation is available through pharmacies [6]. Adopting a similar policy would dramatically increase treatment capacity in the USA and Mexico. In addition to an increase in MAT capabilities, it is essential that physicians receive more comprehensive addiction training during their medical school and residency years. This may require substantial changes to nationwide medical training standards.

Third, geographic “hot spots” of opioid misuse among marginalized populations should be prioritized for the provision of low-threshold and experimental approaches to MAT delivery. Such models are in place in Hong Kong, where methadone is readily available on the day that it is prescribed [6], as well as in Vancouver, Canada [6]. Within the USA, long-term patient-oriented methadone maintenance in a low-threshold model played a central protective factor in limiting the HIV/AIDS epidemic in New York and this model should be considered for scale up [6]. A truly national low-threshold model for MAT in the USA likely requires a further expansion of access to healthcare among marginalized populations to ensure access to the best treatments for OUD. Such a system should be focused on eliminating disparities in care and should incentivize physicians to serve in settings such as federally qualified healthcare centers, which are located in underserved communities [34].

Fourth, policies of drug decriminalization should be considered to reduce the risk that PWID populations will remain “hidden,” less likely to engage in care, and at higher risk of HIV transmission [35]. In order to do this, models of drug decriminalization, some of which have been implemented at the state level (e.g., Proposition 47 in California), should be adopted nationally [36]. In Canada, recent policy changes have allowed for a scale up of supervised injecting facilities (SIF’s), which divert PWID’s away from prison [36]. Mexico’s 2009 *Narcomenudeo* law policy reform, while not fully implemented, suggests how drug decriminalization can be designed to prioritize the prevention of HIV and other drug-related harms [26]. To avoid barriers to adoption of such drug policy reforms, adequate training of law enforcement and other stakeholders should be incorporated into implementation efforts.

Fifth, there must be an overall shift towards more harm reduction-oriented policing practices. In the USA, there have been recent legislative shifts towards less punitive policing, with the former President Obama’s bill expanding access to naloxone [7], the implementation in the Washington State of Good Samaritan Laws aimed at empowering community members to prevent overdose [37], and California’s Proposition 47 sentencing reform, which in 2014, re-classified personal drug possession and use as a misdemeanor rather than a felony [38]. It is important to note, though, that recent rhetorical shifts in prioritizing treatment over enforcement in the USA are likely due to the perception that the opioid crisis is predominantly affecting white communities [34, 39]. It is essential that physicians continue to advocate for harm reduction-oriented policing, and that the narrative that criminalizes urban black and Latino heroin injectors but sympathetically portrays suburban white heroin users, is rewritten [34]. This is particularly important given the incoming U.S. administration’s initial rhetoric regarding the need for supply-side and enforcement-based responses to drug use.

Finally, pharmaceutical companies and academic research institutions should be further engaged in developing novel pharmacotherapies for OUD accessible to a range of populations. Emerging pharmacotherapies include a buprenorphine 6-month implant that is recently approved for the treatment of opioid dependence [40]. Because the implant minimizes the need for frequent follow-up, it may be well suited to a low-threshold model for marginalized or transient patients who interact infrequently with the healthcare system. As novel delivery methods are tested and approved for use, further testing and innovation is needed to ensure the efficacy of new models for vulnerable populations with respect to maximizing access and minimizing risks.

### An emerging paradigm for OUD prevention

For persons who abuse prescription opioids, preventing the transition from oral, intranasal, or smoking use to injection is a key public health priority [36]. It is also known that PWID play a key role in initiating others into drug injecting. Because enrolling PWID in effective addiction treatment reduces the frequency that they inject, this may also reduce the frequency with which they initiate others into drug injecting. As such, Treatment as Prevention (TasP)—a key plank of efforts to curb the global HIV/AIDS pandemic—may be adaptable to responding to the opioid misuse epidemic. Briefly, TasP refers to the phenomenon whereby the provision of antiretroviral therapy for HIV-positive individuals, which dramatically reduces morbidity and mortality associated with HIV disease progression, also contributes to reductions in rates of HIV incidence by reducing individuals’ HIV viral load [41]. An opportunity therefore exists to apply this paradigm to opioid injecting, given the social communicability of injection drug use and the effectiveness of MAT in contributing to reductions in injecting frequency, public injecting, and supporting eventual abstinence. In Switzerland, where MAT and a range of harm reduction interventions were brought to scale in 1993 as part of a public health-oriented drug policy reform, the proportion of recent injectors (i.e., those who had initiated in the past 2 years) among the country’s PWID population dropped from 19% in 1993 to 3% in 2000, suggesting that this period of MAT scale up was associated with a risk environment less conducive to injection initiation [42]. It is also noteworthy in this regard that emerging evidence suggests that among a cohort of PWID in San Diego, California, those with a history of MAT enrollment had a significantly lower risk of reporting initiating others into drug injecting [43]. As such, MAT may have the potential to improve not only individual but also population-level outcomes related to OUD and opioid dependence in particular [36].

## Conclusion

The opioid misuse epidemic is a complex continental issue with important consequences for public health. As North American countries seek to effectively respond to this epidemic, clinicians must support and advocate for the development of an evidence-based addiction treatment system that is accessible to marginalized populations and effective in managing the unacceptably high burden of OUD across the USA, Mexico, and Canada.

## Abbreviations

HIV: Human immunodeficiency virus
MAT: Medication-assisted treatment
OUD: Opioid use disorder
PWID: People who inject drugs
SIF: Supervised injection facility
TasP: Treatment as Prevention
